# Evidence for Retrovirus and Paramyxovirus Infection of Multiple Bat Species in China

**DOI:** 10.3390/v6052138

**Published:** 2014-05-16

**Authors:** Lihong Yuan, Min Li, Linmiao Li, Corina Monagin, Aleksei A. Chmura, Bradley S. Schneider, Jonathan H. Epstein, Xiaolin Mei, Zhengli Shi, Peter Daszak, Jinping Chen

**Affiliations:** 1 Guangdong Entomological Institute, South China Institute of Endangered Animals, Guangzhou 510260, China; E-Mails: yuanlh@gdei.gd.cn (L.Y.); limm0620@126.com (M.L.); lilinmiao-8245524@163.com (L.L.); twenty-five@live.cn (X.M.); Chenjp@gdei.gd.cn (J.C.); 2 Metabiota, San Francisco, CA 94104, USA; E-Mails: cmonagin@metabiota.com (C.M.); bschneider@metabiota.com (B.S.S.); 3 EcoHealth Alliance, New York, NY 10001, USA; E-Mails: chmura@ecohealthalliance.org (A.A.C.); epstein@ecohealthalliance.org (J.H.E.); daszak@conservationmedicine.org (P.D.); 4 Wuhan Institute of Virology of Chinese Academy of Sciences, Wuhan 430071, China; E-Mail: zlshi@wh.iov.cn

**Keywords:** bat, metagenomics, hipposideridae, pathogens, viral emergence, virome, zoonoses

## Abstract

Bats are recognized reservoirs for many emerging zoonotic viruses of public health importance. Identifying and cataloguing the viruses of bats is a logical approach to evaluate the range of potential zoonoses of bat origin. We characterized the fecal pathogen microbiome of both insectivorous and frugivorous bats, incorporating 281 individual bats comprising 20 common species, which were sampled in three locations of Yunnan province, by combining reverse transcription polymerase chain reaction (RT-PCR) assays and next-generation sequencing. Seven individual bats were paramyxovirus-positive by RT-PCR using degenerate primers, and these paramyxoviruses were mainly classified into three genera (*Rubulavirus*, *Henipavirus* and *Jeilongvirus*). Various additional novel pathogens were detected in the paramyxovirus-positive bats using Illumina sequencing. A total of 7066 assembled contigs (≥200 bp) were constructed, and 105 contigs matched eukaryotic viruses (of them 103 belong to 2 vertebrate virus families, 1 insect virus, and 1 mycovirus), 17 were parasites, and 4913 were homologous to prokaryotic microorganisms. Among the 103 vertebrate viral contigs, 79 displayed low identity (<70%) to known viruses including human viruses at the amino acid level, suggesting that these belong to novel and genetically divergent viruses. Overall, the most frequently identified viruses, particularly in bats from the family *Hipposideridae*, were retroviruses. The present study expands our understanding of the bat virome in species commonly found in Yunnan, China, and provides insight into the overall diversity of viruses that may be capable of directly or indirectly crossing over into humans.

## 1. Introduction

Bats (order Chiroptera) comprise the second largest mammalian group in the world and are widely distributed across six continents [1]. Numerous bat species have been implicated as natural reservoirs for significant zoonotic viruses, including Ebola and, Marburg viruses; Nipah and Hendra viruses; SARS coronavirus; and most recently [2,3,4,5]. Collectively, bats harbor a broad diversity of viruses, and it has been proposed that entire viral families and genera, such as pararmyxoviruses, hepaciviruses, and pegiviruses, may have originated from bats [6,7]. More than 170 viruses have been detected in bats, and many of these viruses are highly pathogenic to humans [8], including, Nipah and Hendra viruses [9], rabies virus [10], Australian Bat Lyssavirus [11], Marburg virus [12], and the SARS-like coronavirus [13]. Notably, the outbreak of SARS coronavirus in 2002, which originated in horseshoe bats in China and infected more than 8000 people globally, has spurred increased efforts from the international research community to characterize the diversity and distribution of bat viruses around the world [4,14].

Over 120 bat species have been identified in China, and many are widely distributed throughout the southern provinces of Yunnan, Guangdong, Guangxi, and Fujian [15]. These more ubiquitous bat species, which are in the genera of *Rousettus*, *Myotis*, *Miniopterus* and *Hipposideros*, naturally reside in close proximity to humans, thus increasing the potential of transmission of zoonotic pathogens to humans. Highly pathogenic paramyxoviruses have been identified in a variety of vertebrates, including humans, and phylogenetic reconstruction of host associations suggests a predominance of host switches from bats to other mammals and birds [6], and often cause serious outbreaks of diseases [16,17]. Thus, assessing the variety of paramyxoviruses circulating in bats in China could provide important guidance for the control and prevention of future epidemics.

Recently, high-throughput sequencing (e.g., Illumina, Solexa, *etc*.)—a rapid and efficient technique using sequence-independent amplification of nucleic acids followed by shotgun sequencing—has been employed with great success to discover an enormous diversity of viruses in a range of samples, including marine and fresh water [18,19], animals tissues [20,21], human feces, and bat fecal, urine and oral samples [22,23,24,25,26,27,28,29]. In this study, we used reverse transcription polymerase chain reaction (RT-PCR) to survey the prevalence of paramyxoviruses in bats from Yunnan China, and then conducted Illumina sequencing to characterize paramyxoviruses from bat samples. To date, this is the first reported metagenomic analysis in fecal samples of both insectivorous and frugivorous bats in China.

## 2. Experimental

### 2.1. Ethics Statement

This study was approved by the Guangdong Entomological Institute Administrative Panel on Laboratory Animal Care (Guangzhou, China). All bats were released unharmed immediately after sampling.

### 2.2. Sample Collection and Viral Nucleic Acids Preparation

Between November 2011 and March 2012, a total of 562 oral and rectal swab samples from 281 individual bats of 20 species were collected at multiple sites in the Yunnan Province of China. Of the samples collected, 252 samples (44.84%) were from 126 insectivorous bats and 310 samples (55.16%) from 155 frugivorous bats. The sampling locations include Yuanjiang Gulong hole (N23°35.180', E101°57.540'; H: 405 m), Xishuangbanna Botanical Garden (N: 21°55.368', E: 101°15.235', H: 535 m), and the Natural Arch (N: 21°59.235', E: 101°21.418', H: 919 m). Samples were suspended in a phosphate buffer solution (PBS) with antibiotics and stored at −80 °C until nucleic acid extraction. For the sampling details, such as numbers, locations and specific bat species, see Table S1.

To extract viral nucleic acid, the swab suspensions were centrifuged at 5000× *g* for 10 min. Then, the supernatant was transferred to fresh tubes and centrifuged at 12,000× *g* for 20 min. The viral DNA and RNA were simultaneously extracted from a 140 μL sample with the QIAamp Viral RNA Mini Kit (QIAgen, Düsseldorf, Germany) according to the manufacturer’s protocol and eluted into 60 μL AVE buffer.

### 2.3. Screening of Paramyxoviruses and Phylogenetic Analysis

Sensitive and broadly reactive RT-PCR assays were performed at Wuhan Institute of Virology, Chinese Academy of Sciecnces. In this study, the primers, which were designed according to the conserved motifs of the RNA polymerase (L)-coding sequence, were from the reference Tong *et al.* [30], and could be used for identification of novel paramyxoviruses. The 560 bp fragments of the RNA polymerase (L)-coding sequence conserved in the *Paramyxovirinae* subfamily were amplified, according to the reference Tong *et al.* [30] with no modification. The PCR productions were sequenced using Big Dye Terminator kits (Life Technologies, Carlsbad, CA, USA) on an ABI 3730 automated sequencer with primer PAR-R.

To assess the phylogenetic relationship of paramyxoviruses identified in this study, 79 partial and full L-gene nucleotide sequences were downloaded from NCBI with the GenBank accession numbers listed in Table S2. Nucleotide sequences were then aligned and translated by using ClustalX 1.83 [31]. The best-fit model (GTR + I + G) of the phylogenetic relationship was determined by Modeltest 3.7 [32] and the phylogenetic trees of L-gene were constructed by MrBayes 3.1.1 [33], with the sequences of *Pneumovirinae* subfamily as an outgroup.

### 2.4. High-throughput Sequencing and Pathogen Analysis

To further characterize the co-infected viruses in bat paramyxovirus-positive samples, Illumina high-throughput sequencing was conducted. Total nucleic acids were extracted as described above. Viral nucleic acid samples were then pooled, and a 3 μg pooled sample was used for sequencing library preparation. The synthesis of first and second-strand cDNA from the viral RNA was performed using random oligonucleotides/SuperScript II and DNA Polymerase I/RNase H, respectively. After the 3'-end adenylation, DNA fragments were ligated with Illumina PE adapters on both sides to purify and selectively enrich the 200 bp fragments using the AMPure XP system (Beckman Coulter, Fullerton, CA, USA) and Illumina PCR Primer Cocktail in a 10 cycle PCR reaction. Finally, a sequencing library was generated by Illumina TruSeq^TM^ RNA Sample Preparation Kit (Illumina, San Diego, CA, USA) following the manufacturer’s recommendations and four index codes were added to attribute sequences. The clustering of the index-coded samples was performed on a cBot Cluster Generation System using TruSeq PE Cluster Kit v3-cBot-HS (Illumina, Inc.) according to the manufacturer’s instructions. After cluster generation, the library preparations were sequenced on an Illumina Hiseq 2000 platform and 100 bp paired-end reads were generated. Reads that were contaminated with adapter or of low quality, as well as poly-N reads, were removed from raw data and clean reads were obtained.

### 2.5. Identification of Viral Homologous Sequences

Clean reads from Illumina were compiled with *de novo* assembly, using de Bruijn graphs assembly algorithms [34], and contigs <200 bp in length were not analyzed further. Remaining sequences underwent sequential BLASTx searching against GenBank database to eliminate the bacteria and eukaryotes contigs and identify suspect-viral sequences, which were longer than 200 bp, and taxonomic classification was queried from the NCBI taxonomy web service. The reference sequence of the source organism with the best contig alignment (*i.e.*, the alignment with the lowest e-value ≤ 0.0001) was retrieved. Contigs of bacteria and eukaryotes were eliminated. If the reference sequence taxonomy was viral and aligned with the contig with an e-value of ≤0.0001, the sequence was flagged as suspect-viral and retained for further analysis.

### 2.6. Phylogenetic Analysis of Viral Sequences

Suspect viral sequences related to invertebrate virus families were excluded. Reference sequences were downloaded from NCBI. Global alignments with contigs were generated using ClustalX 1.83 [31], translated in MEGA 5 [35] and edited with BIOEDIT v7.0 [36]. Gap-stripped alignments were then used to construct the phylogenetic trees by MrBayes 3.1.1 [33].

### 2.7. Nucleotide Sequence Accession Numbers

The Illumina sequence data obtained in this study have been deposited in the Sequence Read Archive (SRA) database (Accession No. SRX368740). The trimmed and binned vertebrate viral contigs (≥200 bp) used for phylogenetic analysis in this study were deposited in GenBank (Accession No. KF547868-KF547871).

## 3. Results

### 3.1. Detection of Paramyxoviruses

Among the samples, seven fecal samples of seven individual bats were identified as paramyxovirus-positive, and in these bats, two individuals belong to *Hipposideros cineraceus* (sampled in the Yuanjiang Gulong hole), one of *Rousettus leschenaultii*, one of *Eonycteris spelaea*, one of *Hipposideros armiger* (sampled from the Xishuangbanna Botanical Garden, Mengla, China), and three *Taphozous melanopogon* (sampled in Natural Arch, Mengla, China) (Table S1). The ~560-bp L-gene sequences were submitted to GenBank (Accession No. KC599255, KC599257- KC599261, KC599263).

**Figure 1:**
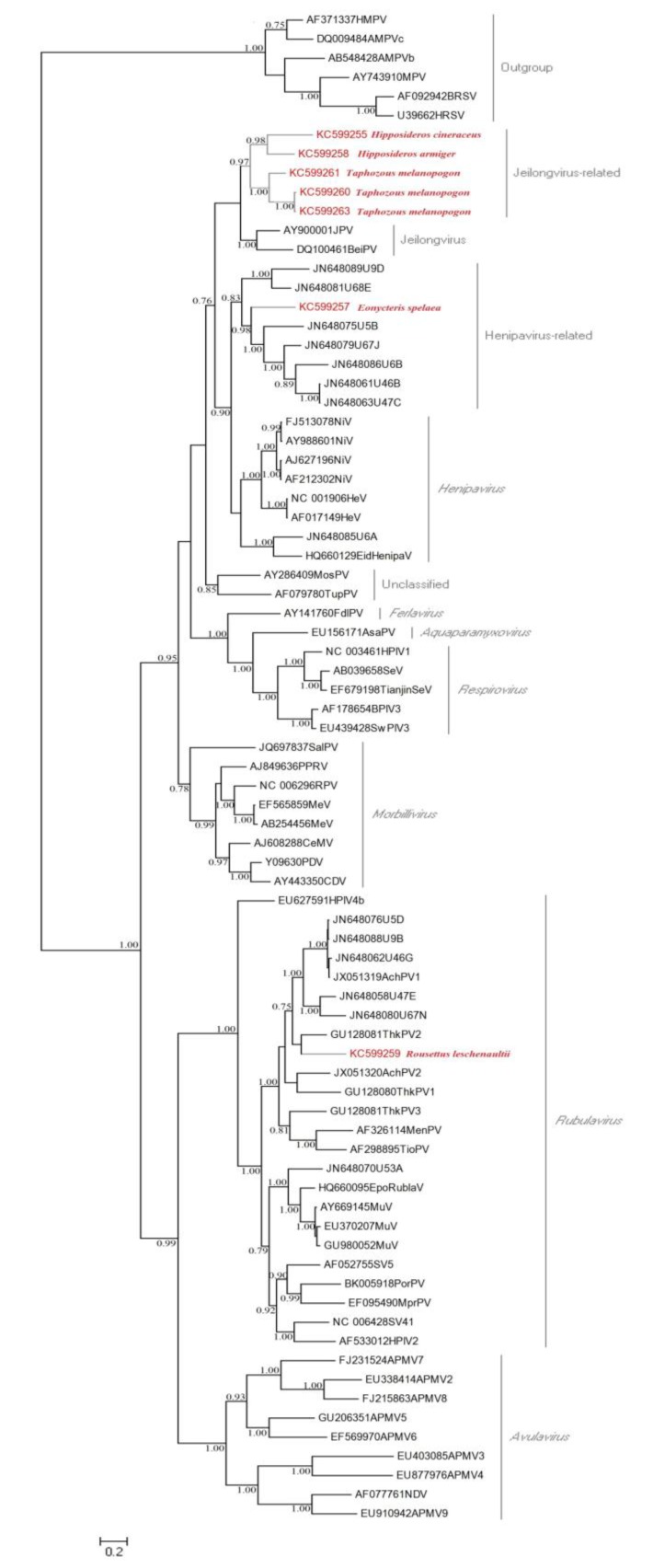
Phylogenetic analysis of paramyxovirus L-gene identified by RT-PCR. Phylogenetic tree of paramyxoviruse L-gene was constructed based on a 529 bp sequence alignment with *Pneumovirinae* subfamily as an outgroup. The accession numbers of sequences identified in this study are red and posterior probability values are shown next to the tree nodes. Posterior probability values are shown at each node (>70%) and the bar represents the expected number of amino acid substitutions per site.

The phylogenetic tree of the L-gene, based on a 529 bp alignment is shown in Figure 1. The phylogenetic analysis indicates that the paramyxovirus sequences identiﬁed in this study are separated into three distinct genera. Of them, KC599259 identified in *R. leschenaultii* was clustered with *Rubulavirus*. KC599257 detected in *E. spelaea* showed a close relationship with paramyxoviruses from *Eidolon helvum* in urban Africa, which formed an unclassified sister clade to the genus *Henipavirus.* The third cluster is that of five novel paramyxoviruses sharing a common ancestor and forming a phylogenetically diverse subgroup, closely related to genus *Jeilongvirus*, comprising Jeilongvirus (J-virus) and Beilong virus [37]. Moreover, this cluster can be further divided into two clades, KC599255 and KC599258 from *Hipposideridae*; and KC599260, KC599261, and KC599263 from *T. melanopogon* (family *Emballonuridae*). The deduced amino acid identity analysis supported the phylogenetic relationship observed using the nucleotide sequences (Table S3). KC599259 had the lowest homology (37.58%–40.61%) with other paramyxovirus sequences identified in this study and had a higher identity (61.49%–78.74%) with known *Rubulavirus* and *Rubulavirus*-related virus. The novel paramyxoviruses (KC599255, KC599258, KC599260, KC599261 and KC599263) from insectivorous bats shared the highest amino acid identities (74.55%–100%), and had a relatively high homology with Jeilongvirus (74.25%–78.44%) and KC599257 (59.39%–64.85%). On the other hand, KC599257 was most closely related to Henipaviruses (92.94%).

### 3.2. In-Depth Analysis of Pathogens by Illumina High-throughput Sequencing

**Figure 2:**
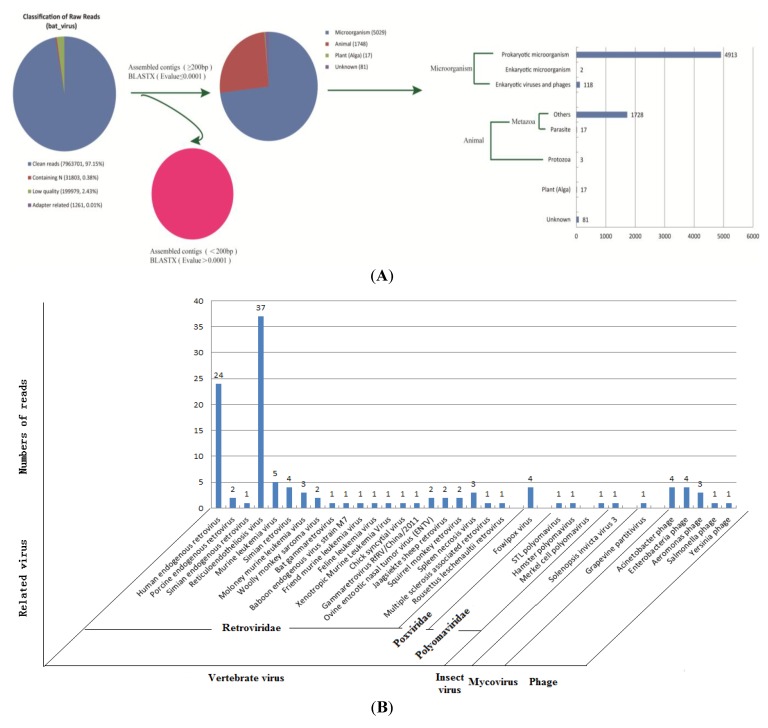
Schematic summary of the number of Illumina high-throughput sequencing reads. (**A**) Classification of raw reads, and contigs (≥200 bp) compared with Genbank using BLASTx searches (e-value < 0.0001); (**B**) Reads related to eukaryotic viruses and phages.

Individual sequence reads with base quality scores were produced by Illumina. After removing the contaminant reads (0.01% the adapter reads, 2.43% low quality reads and 0.38% containing N reads), a total of 7,963,701 clean reads (97.15%) were obtained in this study (Figure 2A).

#### 3.2.1. Assembled Contigs and BLASTx Analysis

A total of 7066 assembled contigs (≥200 bp) were constructed and 6880 contigs with e-value ≤ 0.0001 were obtained. Data showed that the longest contig was 6481 bp and 310 contigs were longer than 1000 bp. A total of 73.1% (5029/6880) of contigs associated with microorganisms. 118 contigs that were homologous to eukaryotic viruses and phage. Moreover, we found 17 contigs that were homologs of parasites (Table 1, Figure 2, Table S4 and Table S5).

**Table 1:** Details of assembled contigs related to eukaryotic viruses and phages as determined with Blastx and the GenBank database. Detailed information is shown in Table S6.

Clade	Family	Genus	Virus Name	Contigs
Vertebrate virus	Retroviridae	Gammaretrovirus	Human endogenous retrovirus	24
			Murine leukemia virus	5
			Spleen necrosis virus	4
			Moloney murine leukemia virus	3
			Porcine endogenous retrovirus	2
			Woolly monkey sarcoma virus	2
			Friend murine leukemia virus	1
			Feline leukemia virus	1
			Xenotropic Murine Leukemia Virus	1
			Chick syncytial virus	1
			Gammaretrovirus RfRV/China/2011	1
		Unknown gammaretrovirus	Reticuloendotheliosis virus	40
			Bat gammaretrovirus	1
			Baboon endogenous virus strain M7	1
			Rousettus leschenaultii retrovirus	1
		Betaretrovirus	Ovine enzootic nasal tumor virus (ENTV)	2
			Jaagsiekte sheep retrovirus	2
			Squirrel monkey retrovirus	2
			Simian endogenous retrovirus	1
		Unclassified	Simian retrovirus	4
			Multiple sclerosis associated retrovirus	1
	Polyomaviridae	Polyomavirus	STL polyomavirus	1
			Hamster polyomavirus	1
			Merkel cell polyomavirus	1
Insect virus			Solenopsis invicta virus 3	1
Mycovirus			Grapevine partitivirus	1
Phage	Myoviridae	T4-like virus	Acinetobacter phage Ac42	1
	Myoviridae	T4-like virus	Acinetobacter phage Acj61	1
	Myoviridae	T4-like virus	Acinetobacter phage Acj9	2
	Myoviridae	T4-like virus	Aeromonas phage 44RR2.8t	1
	Myoviridae	T4-like virus	Aeromonas phage Aeh1	1
	Myoviridae	T4-like virus	Aeromonas phage Aes508	1
	Myoviridae	T4-like virus	Enterobacteria phage Bp7	1
	Myoviridae	T4-like virus	Enterobacteria phage JSE	1
	Myoviridae	T4-like virus	Enterobacteria phage Phi1	1
	Myoviridae	T4-like virus	Enterobacteria phage RB69	1
	Myoviridae	unclassified Myoviridae	Salmonella phage STML-198	1
	Myoviridae	unclassified Myoviridae	Yersinia phage phiR1-RT	1

#### 3.2.2. Identification of Bacteriophage Sequences

There were 13 contigs related to the sequences of known phages with a similarity of 61%–89% and an e-value of 6*e*^−71^–2.12*e*^−5^. All of them were members of *Myoviridae* and 11 contigs were related to T4-like entero-bacterial phages (Table 1, Figure 2B and Table S5). 

#### 3.2.3. Analysis of Vertebrate Viral Sequences by Family

Thirteen contigs of phage, 1 insect-virus, and 1 mycovirus contig were removed, and 103 contigs belonging to two mammalian-infecting viral families were left. Of them, 97% (100/103) were related to retroviruses and three contigs were related to a polyomavirus of double-stranded DNA (dsDNA). For the 103 contigs of vertebrate viruses, the amino acid sequences were 35% to 90% homologous with viral protein sequences (e-value ≤ 0.0001). In these, 79 cotigs displayed low identity (<70%) to known viruses at the amino acid level, suggesting that these belong to novel and genetically divergent viruses. The nucleotide BLASTx searches against the GenBank database are listed in Table S6.

#### 3.2.4. Retroviridae

**Figure 3:**
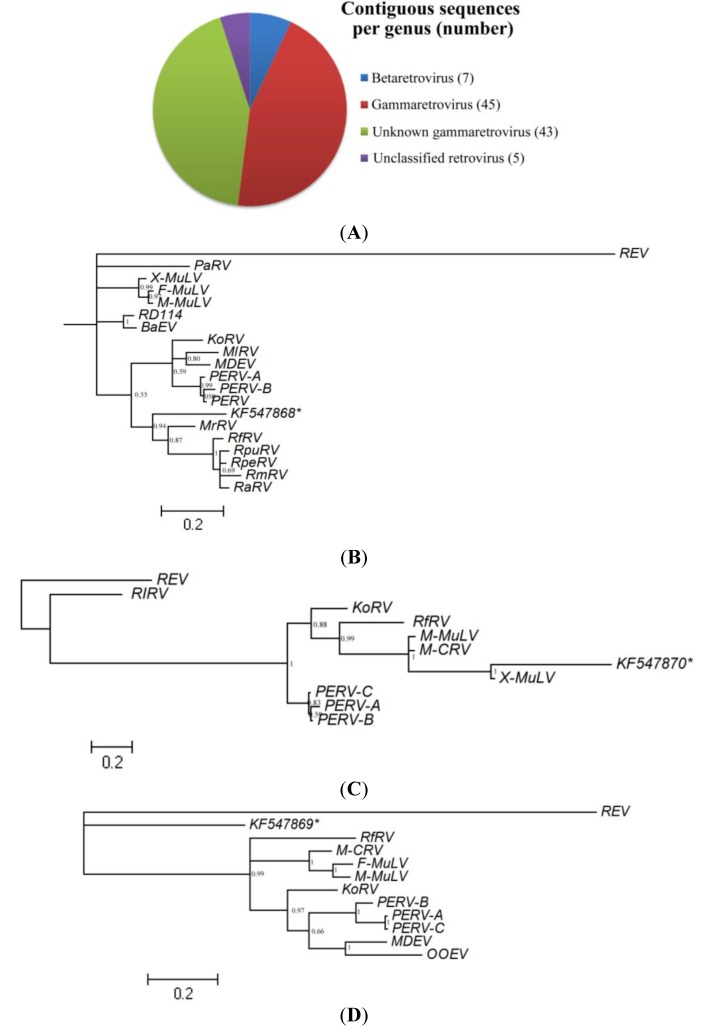
Classification and phylogenetic analysis of retroviral contigs. (**A**) The chart shows proportions of retroviral sequences related to different retroviral genera. The number of sequences related to each genus is shown in parentheses; (**B**)–(**D**) The midpoint-rooted phylogenetic tree based on alignments of 83 aa Pol (starred), 142 aa Gag (starred) and 150 aa Env (starred) of bat retroviruses, respectively. Posterior probability values are shown at each node and the bar represents the expected number of amino acid substitutions per site. Sequence information is shown in Table S7.

We identified 100 contigs, primarily related to three genera of retroviruses (Gamma, beta and unclassified retroviruses), and with e-values of 5*e*^−117^–1*e*^−4^ (Figure 3A, also see Table S6). These retroviral sequences related to all three canonical genes of retrovirus, including 28 Gag proteins, 66 protease/polymerase (Pol) and 6 envelope glycoproteins (Env), and some of them are highly closed to known bat tretroviruses [38,39,40]. Three retrovirual contigs, which code Pol, Gag and Env of gammaretrovirus respectively and have the longest CDS, were used for phylogenetic analysis. Due to the amplification of the short retroviruses sequences, we have adjusted the tree according to the published bat retrovirus trees [39,40] by removing some of the reference sequences. The phylogenetic trees indicated that Pol (KF547868) and Gag (KF547870) showed high homologous with bat gammaretroviruses, whereas Env (KF547869) couldn’t clade with those of bat gammaretroviruses indicating the possibility of new viruses (Figure 3B–D). Moreover, sequence analysis indicated that the translations of 55 of the 100 retrovirus contigs contained stop codons within the region of BLASTx alignments, suggesting that they originated from non-functional, endogenous/exogeneous retroviruses. In addition, BLASTx searching indicated that many contigs (such as comp6291_c0_seq1, comp48905_c0_seq1, comp8098_c0_seq1, comp6984_c0_seq1, comp92776_c0_seq1, comp71626_c0_seq1 and comp36376_c0_seq1) shared equivalent or relatively higher amino acid identity (52%–85%, e-values of 7*e*^−12^–4*e*^−35^) with poxvirus rather than retroviruses, with Fowlpox virus (FPV) being the closest relative. 

#### 3.2.5. Polyomaviridae

**Figure 4:**
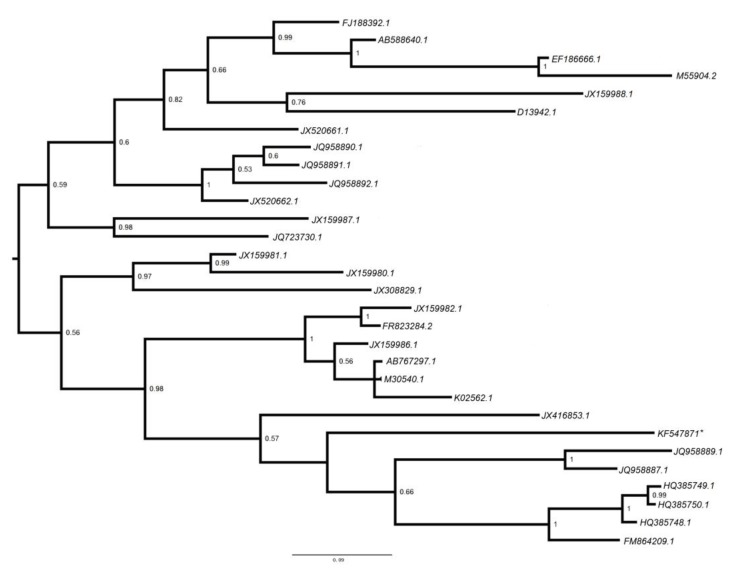
Phylogenetic analysis of polyomaviral contig. The midpoint-rooted phylogenetic tree based on 111 aa VP1 protein of polyomavirus (starred). Posterior probability values are shown at each node and the bar represents the expected number of amino acid substitutions per site.

We assembled two sequences related to the VP1 capsid protein and a sequence related to the large T antigen protein of polyomaviruses (Table S6). They are phylogenetically clustered with hamster polyomavirus, merker cell polyomavirus and STL polyomavirus respectively, and with high confidence of e-values of 2*e*^−15^–2*e*^−53^ (Table S6 and Figure 4).

## 4. Discussion

With the increase of human activities, habitat and survival of bats are severely reduced, thus causing habitat shifts in bat populations and increased opportunity for contact with humans. The close contact between humans and bats expands the opportunity for cross-species transmission of new zoonotic viruses to humans [41]. In the current study, we identified seven bats [including three insectivorous bats (*Hipposideros cineraceus*, *H. armiger* and *Taphozous melanopogon*) and two frugivorous bats (*Eonycteris spelaea* and *Rousettus leschenaultii*)] positive for paramyxovirus by RT-PCR. Phylogenetic analysis indicated that paramyxoviruses carried by insectivorous bats were distinct from those found in frugivorous bats (Figure 1). Virus sequences from insectivorous bats could not be definitely assigned to any existing paramyxovirus genus, however, they appeared to cluster within the relatively new genus of the *Jeilongvirus* [37]. Moreover, we found that KC599257 (*E. spelaea*) and KC599259 (*R. leschenaulti*) from fruit bats belonged to *henipavirus*-related paramyxovirus and *Rubulavirus* genus, respectively. The finding of a henipavirus-related agent in *E. spelaea* provides further evidence that there is a large spectrum of henipa-like viruses among bats, including insectivorous species. Li *et al.* [42] previously found serological evidence of henipa-like viruses in bats, including *Rousettus spp.*, *Myotis spp*., *Hipposideros spp.*, and *Miniopterus spp.* in China. Henipaviruses have thus far overwhelmingly been identified in *Pteropus* and *Eidolon* species, though this may, in part, be due to a lack of surveillance in non-pteropodid bats [2,17,43]. The phylogenetic distance of species may constrain both cross-species transmission and host shifts and thus result in the separation of viruses [44]. Previous studies demonstrate that humans can be infected by paramyxoviruses through a secondary host species, such as horses and pigs, or directly from bats [9,45,46,47]. Although it is currently unknown whether paramyxoviruses, such as Jeilongvirus, are capable of infecting people, the potential risks of bat borne paramyxoviruses to human health should be considered and surveys of human populations highly exposed to bats for evidence of spillover of bat paramyxoviruses would help establish the public health risk.

High-throughput sequencing-based viral metagenomics is a powerful tool to explore known and unknown viruses existing in host animals. Recently, it has been successfully used to detect viruses in bats in North America, Africa and Asia [22,23,26,27,28], where the viromes of one frugivorous bat species, *E. helvum* in Africa, and several insectivorous bats were described. From our metagenomic study of paramyxovirus-positive bat samples, full-length viral genomic sequences were unable to be assembled from the clean reads. This result is similar with that of previous studies and may be due to the low quantity of the virus within samples, the short reads, and the lack of known reference sequences [22,23,26]. Previous studies demonstrated that protein-based comparisons are more effective than those based on nucleotides, and thus BLASTx usually identifies more suspect-viral sequences than BLASTn [28,48]. Among the viral contigs, most of the bacterioviruses detected in this study are enterobacterial phages, suggesting the similarity between the intestinal bacterial population of bats and that of humans. Five contigs are highly similar to Moloney Murine Leukemia Virus (M-MLV), which may have resulted from contamination of RT reagents and also have been detected in previous virome analysis of insectivorous bats in China [26]. Many contigs are closed to bat retrovirues, which were identified by previous studies [38,39,40]. Furthermore, we found that many viral contigs showed equivalent or higher identity with Fowlpox virus (FPV) more than Reticuloendotheliosis virus (REV). As a prototypical member of *Avipoxvirus*, FPV only infects non-mammalian hosts [49], and there is no previous data available to indicate that bats are natural reservoirs or vectors of FPV. Previous studies show that REVs derive directly from mammalian retroviruses and integrate into FPV genomes, which become endogenous in the genome of larger and more complex DNA viruses [50,51,52]. Thus, these FPV-like contigs were deemed as REVs and classified into unknown gammaretroviruses temporarily, and the source of FPV-like contigs in this study should be explored further.

Our sequence analysis indicates that majority of the vertebrate viral sequences were distinct from those previously identified in bats and were often diverse within the viral family. The novel viruses identified were classified into two viral families, *Retroviridae* and *Polyomaviridae*, which were also detected in previous studies [26,27,28]. Retroviruses have both endogenous and exogenous forms in nature, comparing the results from this study with previous studies, metagenomic virome analysis indicated that retroviruses are the most common viral sequences found in bats, especially in *Hipposideridae* [26,29]. This finding may be due to the frequent integration of retroviral genomic material into host genomes, rather than an indication of viral infection. Interestingly, the Illumina screening did not detect paramyxovirus nucleic acid, which had been detected by RT-PCR, suggesting that these viruses may be present in samples in relatively small amounts and below the sensitivity of Illumina detection. These findings highlight an important limitation of high throughput sequencing as a tool for virome characterization. Furthermore, the limited size or number of contigs generated by Illumina sequencing may necessitate follow-up with conventional PCR assays in order to obtain enough sequence to perform meaningful phylogenetic analyses [6,28]. The sensitivity of high-throughput sequencing to detect viral nucleic acid is also limited if samples are not properly filtered and treated to remove host genome and other non-target nucleic acid and enrich for viral sequences. Host nucleic acid are typically present in significantly higher abundance relative to viral nucleic acid and can create background noise in the results [26]. As the cost of high-throughput sequencing decreases and methods of sample preparation improve to optimize results, Illumina sequencing can be an effective tool for characterizing virus diversity in host organisms. Bats represent a massively diverse group of mammals, and as a group they have been increasingly identified as natural reservoirs for virus families such as paramyxoviruses, coronaviruses, and retroviruses, that contain zoonotic members. Emerging zoonotic bat-borne viruses, such as SARS coronavirus; Nipah and Hendra virus; and Marburg and Ebola virus; illustrate that bat viruses are capable of infecting people through various routes of transmission and in various contexts such as through domestic animals, food-borne routes, or the wildlife trade. Understanding the underlying diversity of viruses within common bat species and the types of interactions they have with people, wildlife and livestock in China may provide further insight into the risk of viral spillover, and may ultimately allow for interventions that reduce the risk of outbreaks.

## 5. Conclusions

We, for the first time, carry out the metagenomic analysis in fecal samples of both insectivorous and frugivorous bats in China. The present study contributes to the understanding of the bat virome, and sheds light on the overall diversity of zoonotic viruses.

## Supplementary Files

Supplementary File 1 Supplementary Information (PDF, 928 KB)

Supplementary File 1 Supplementary Table (XLS, 62 KB)
