# Supplementary material for: Evidence for Retrovirus and Paramyxovirus Infection of Multiple Bat Species in China
Source: Viruses. 2014 May 16;6(5):2138–54. doi: 10.3390/v6052138 (PMC4036550; doi:10.3390/v6052138)
Supplement: Supplementary File 1 — Supplementary Information (PDF, 928 KB) [file viruses-06-02138-s001.pdf]

Supplementary for Evidence for Retrovirus and Paramyxovirus Infection of Multiple Bat Species in China

Table S1. Sample information of 20 bat species tested for paramyxoviruses.

Bat species	Bat family	Roosting sites	Sampling site	Total tested		Total positive (n/r)
Rhinolophus pusillus	*Rhinolophidae*	Cave	B/C	1	9	0/0
Rhinolophus affinis		Cave	B	1		
Rhinolophus huananus		Cave	B	1		
Rhinolophus yunanesis		Cave	B/C	6		
Taphozous melanopogon	*Emballonuridae*	Cave	B/C	34	36	3/8.33%
Taphozous perforatus		Cave	C	2		
Eonycteris spelaea	*Pteropodidae*	Tree	C	23	155	1/4.35%
Macroglossus sobrinus		Cave	A	3		
Cynopterus sphinx		Tree	B	78		
Rousettus leschenaulti		Cave	B	51		1/1.96%
Hipposideros cineraceus	*Hipposideridae*	Cave	B	17	27	1/5.88%
Hipposideros larvatus		Cave	A/B	7		
Hipposideros armiger		Cave	B	2		1/50%
Hipposideros pomona		Cave	B/C	1		
Chaerephon plicata	*Molossidae*	Cave	A	7	7	0/0
Pipistrellus abramus	*Vespertilionidae*	House	C	2	47	0/0
Pipistrellus pipistrellus		House	B	1		
Tylonycteris pachypus		Tree	B	6		
Scotophilus heathi		Tree	B	28		
Miniopterus schreibersii		Cave	B	10		
					281	7/2.49%

Cave, Tree and House are all near to humans' habitation and there are close relationships with human life. A: Yuanjiang cologne hole; B: Xishuangbanna botanic garden; C: Natural arch; n: the number of positive bat individuals; r: rate of positive bat individuals.

Table S2. Paramyxovirus available in Genebank used for phylogenetic analyses.

Virus	abbreviation	Genus	Accession Number	host	Country
Nipah virus	AF212302NiV	Henipavirus	AF212302	Pteropus hypomelanus	Malaysia
Hendra virus	AF017149HeV	Henipavirus	AF017149	Pteropus poliocephalus	Australia
Nipah virus	FJ513078NiV	Henipavirus	FJ513078	Homo sapiens	India
Nipah virus	AJ627196NiV	Henipavirus	AJ627196	Sus scrofa	Malaysia
Nipah virus	AY988601NiV	Henipavirus	AY988601	Homo sapiens	Bangladesh
Hendra virus	NC_001906HeV	Henipavirus	NC_001906	Horse	Australia
Eidolon helvum Henipavirus	HQ660129Eid HenipaV	Henipavirus	HQ660129	Eidolon helvum	Ghana
Eidolon helvum PV	JN648075U5B	Henipavirus-related	JN648075	Eidolon helvum	Ghana
Eidolon helvum PV	JN648086U6B	Henipavirus-related	JN648086	Eidolon helvum	Ghana
Eidolon helvum PV	JN648061U46B	Henipavirus-related	JN648061	Eidolon helvum	Ghana
Eidolon helvum PV	JN648063U47C	Henipavirus-related	JN648063	Eidolon helvum	Ghana
Eidolon helvum PV	JN648079U67J	Henipavirus-related	JN648079	Eidolon helvum	Ghana
Human parainfluenza virus 4b	EU627591HPIV4 b	Rubulavirus	EU627591	Homo sapiens	Canada
Simian virus 5	AF052755SV5	Rubulavirus	AF052755	Chlorocebus sabaeus	USA

Table S2. *Cont.*

Virus	abbreviation	Genus	Accession Number	host	Country
Simian virus 41	NC_006428SV41	Rubulavirus	NC_006428	Chlorocebus sabaeus	Japan
Menangle virus	AF326114MenPV	Rubulavirus	AF326114	Pteropus alecto	Australia
Mapuera virus	EF095490MprPV	Rubulavirus	EF095490	Sturnira lilium	Brazil
Mumps virus	EU370207MuV	Rubulavirus	EU370207	Homo sapiens	Croatia
Mumps virus	GU980052MuV	Rubulavirus	GU980052	Homo sapiens	United Kingdom
Mumps virus	AY669145MuV	Rubulavirus	AY669145	Homo sapiens	Russia
Porcine rubulavirus	BK005918PorPV	Rubulavirus	BK005918	Sus scrofa	America
Tioman virus	AF298895TioPV	Rubulavirus	AF298895	Pteropus hypomelanus	Malaysia
Human parainfluenza virus 2	AF533012HPIV2	Rubulavirus	AF533012	Homo sapiens	USA
Tuhoko viruses 1	GU128080ThkPV1	Rubulavirus	GU128080	Rousettus leschenaulti	China
Tuhoko viruses 2	GU128081ThkPV2	Rubulavirus	GU128081	Rousettus leschenaulti	China
Tuhoko viruses 3	GU128082ThkPV3	Rubulavirus	GU128082	Rousettus leschenaulti	China
Achimota viruses 1	JX051319AchPV1	Rubulavirus	JX051319	Eidolon helvum	Ghana
Achimota viruses 2	JX051320AchPV2	Rubulavirus	JX051320	Eidolon helvum	Ghana
Epomophorus species Rubulavirus	HQ660095Epo RublaV	Rubulavirus	HQ660095	Epomophorus species	Congo
Eidolon helvum PV	JN648076U5D	Rubulavirus-related	JN648076	Eidolon helvum	Ghana
Eidolon helvum PV	JN648088U9B	Rubulavirus-related	JN648088	Eidolon helvum	Ghana
Eidolon helvum PV	JN648062U46G	Rubulavirus-related	JN648062	Eidolon helvum	Ghana
Eidolon helvum PV	JN648058U47E	Rubulavirus-related	JN648058	Eidolon helvum	Ghana
Eidolon helvum PV	JN648070U53A	Rubulavirus-related	JN648070	Eidolon helvum	Ghana
Eidolon helvum PV	JN648080U67N	Rubulavirus-related	JN648080	Eidolon helvum	Ghana
Peste-des-petits-ruminants virus	AJ849636PPRV	Morbillivirus	AJ849636	Ovis aries	Turkey
Cetacean morbillivirus	AJ608288CeMV	Morbillivirus	AJ608288	Delphinus delphis	United Kingdom
Measles virus	EF565859MeV	Morbillivirus	EF565859	Homo sapiens	France
Measles virus	AB254456MeV	Morbillivirus	AB254456	Homo sapiens	Japan
Rinderpest virus	NC_006296RPV	Morbillivirus	NC_006296	Bos auben	Kenya
Phocine distemper virus	Y09630PDV	Morbillivirus	Y09630	Phoca vitulina	United Kingdom
Canine distemper virus	AY443350CDV	Morbillivirus	AY443350	Procyon lotor	USA
Sendai virus	AB039658SeV	Respirovirus	AB039658	Mus musculus	Japan
Tianjin Sendai virus	EF679198Tianjin SeV	Respirovirus	EF679198	Callithrix jacchus	China
Bovine parainfluenza virus 3	AF178654BPIV3	Respirovirus	AF178654	Bovine	USA

Table S2. *Cont.*

Virus	abbreviation	Genus	Accession Number	host	Country
Human parainfluenza virus 1	NC_003461HPIV1	Respirovirus	NC_003461	Homo sapiens	USA
Sus scrofa parainfluenza virus 3	EU439428SwPIV3	Respirovirus	EU439428	Sus scrofa	USA
Newcastle disease virus	AF077761NDV	Avulavirus	AF077761	Gallus gallus	Netherlands
Avian paramyxovirus 2	EU338414APMV-2	*Avulavirus*	EU338414	Gallus gallus	USA
Avian paramyxovirus 3	EU403085APMV-3	*Avulavirus*	EU403085	Meleagris gallopavo	Netherland
Avian paramyxovirus 4	EU877976APMV-4	*Avulavirus*	EU877976	Anas platyrhynchos	South Korea
Avian paramyxovirus 5	GU206351APMV-5	*Avulavirus*	GU206351	Melopsittacus aubenton	Japan
Avian paramyxovirus 6	EF569970APMV-6	Avulavirus	EF569970	Anas domesticus	Russia
Avian paramyxovirus 7	FJ231524APMV-7	Avulavirus	FJ231524	Columbidae species	USA
Avian paramyxovirus 8	FJ215863APMV-8	Avulavirus	FJ215863	Branta aubentoni	USA
Avian paramyxovirus 9	EU910942APMV-9	Avulavirus	EU910942	Anas domesticus	USA
Atlantic salmon paramyxovirus	EU156171AsaPV	Aquaparamyxovirus	EU156171	*Salmo salar*	Norway
Fer-de-Lance paramyxovirus	AY141760FdlPV	Ferlavirus	AY141760	Fer-de-Lance Viper	USA
Mossman virus	AY286409MosPV	Undefined	AY286409	wild rat	Australia
Salem virus	JQ697837SalPV	Undefined	JQ697837	horse	USA
Tupaia paramyxovius	AF079780TupPV	Undefined	AF079780	Tupaia belangeri	Germany
J-virus	AY900001JPV	Undefined	AY900001	Mus musculus	Australia
Beilong virus	DQ100461BeiPV	Undefined	DQ100461	Rattus norvegicus	China
Eidolon helvum PV	JN648085U6A	Undefined	JN648085	Eidolon helvum	Ghana
Eidolon helvum PV	JN648089U9D	Undefined	JN648089	Eidolon helvum	Ghana
Eidolon helvum PV	JN648081U68E	Undefined	JN648081	Eidolon helvum	Ghana
Human metapneumovirus	AF371337HMPV	Metapneumovirus	AF371337	Homo sapiens	Netherlands
Avian metapneumovirus C	DQ009484AMPV-C	Metapneumovirus	DQ009484	Branta aubentoni	USA
Avian metapneumovirus B	AB548428AMPV-B	Metapneumovirus	AB548428	Meleagris gallopavo	France
Murine pneumonia virus	AY743910MPV	Pneumovirus	AY743910	Mus musculus	USA
Bovine respiratory syncytial virus	AF092942BRSV	Pneumovirus	AF092942	Bovine kidney cell	Germany
Human respiratory syncytial virus S2	U39662HRSV	Pneumovirus	U39662	Homo sapiens	UK

Abbreviation represents virus sequences in the phylogenic tree.

Table S3. Comparision of amino acid identities.

		YN12137	YN12069	YN12162	YN12193	YN12167	YN12003	YN12103
YN12137		100						
YN12069		39.39	100					
YN12162		38.18	63.64	100				
YN12193		38.18	63.64	100	100			
YN12167		37.58	61.82	96.36	96.36	100		
YN12003		40.61	59.39	75.76	75.76	74.55	100	
YN12103		38.18	64.85	78.79	78.79	79.39	83.03	100
MenPV		67.24	33.53	32.92	33.72	33.94	36.88	36.36
TioPV		63.22	32.94	32.3	33.72	33.94	33.12	34.66
ThkPV1		65.52	31.76	34.78	36.05	35.15	35	34.66
ThkPV2		78.74	35.88	32.3	33.14	33.33	35.62	34.09
ThkPV3		72.41	35.88	32.92	34.88	33.94	35.62	35.8
AchPV1	R	72.41	32.94	35.23	33.14	35.15	36.25	35.23
AchPV2	u	74.71	35.88	32.3	33.72	35.15	36.88	38.07
HPIV4b	b	62.64	37.06	37.27	35.47	35.76	36.88	38.07
SV5	u	64.94	35.88	32.92	38.37	40.61	38.12	38.07
SV41	l	58.05	34.71	34.78	34.3	32.73	34.38	33.52
MuV	a	63.22	37.06	36.65	37.21	38.79	38.12	37.5
MprPV	v	63.79	35.88	34.78	36.05	36.97	35	35.23
PorPV	i	62.07	35.29	32.92	34.3	34.55	33.12	34.09
U5D	r	71.84	32.94	32.3	33.14	35.15	36.88	35.23
U9B	u	72.41	32.94	32.3	33.14	35.15	36.25	35.23
U46G	s	70.11	32.35	32.3	33.14	35.15	36.25	35.23
U47E		68.39	32.94	33.54	34.3	34.55	34.38	34.09
U53A		61.49	34.71	34.78	35.47	36.97	36.25	35.8
U67N		68.39	32.94	32.92	33.72	33.94	33.75	33.52
EpoRublaV		62.07	34.71	36.65	37.21	38.79	37.5	37.5
NC_001906HeV		37.06	65.88	61.49	61.49	59.63	58.12	60.62
AF017149HeV		37.06	65.88	61.49	61.49	59.63	58.12	60.62
FJ513078NiV		38.24	66.47	63.35	63.35	62.73	59.38	62.5
AY988601NiV	H	38.24	66.47	63.35	63.35	62.73	59.38	62.5
AJ627196NiV	e	37.65	65.88	63.98	63.98	63.35	58.75	61.87
AF212302NiV	n	37.65	65.88	63.98	63.98	63.35	58.75	61.87
U5B	i	38.24	67.65	60.87	60.87	60.87	58.75	61.87
U6A	p	35.88	92.94	55.9	55.9	56.52	57.5	58.75
U6B	a	41.76	68.24	57.76	57.76	57.14	56.25	58.75
U9D	v	38.82	70	60.25	60.25	60.87	60	62.5
U46B	i	40	65.88	62.11	62.11	60.25	60	60
U47C	r	40	65.88	62.11	62.11	60.25	60	60
U67J	u	41.76	70.59	60.87	60.87	59.63	59.38	60.62
U68E	s	41.18	70.59	62.73	62.73	59.63	65.62	66.25
EidHenipaV		37.06	62.94	57.14	57.14	57.14	56.25	58.12
JPV		40.61	61.08	76.65	77.25	76.05	74.25	78.44
BeiPV		40.61	59.28	74.85	75.45	76.05	76.05	75.45

Table lists percentage of identity between deduced amino acid sequences from partial L gene fragments identified in the present study and homologous sequences from selected known paramyxoviruses.

Table S4.

**Total contigs (≥200 bp)**	**7066**
e-value($p \leq 0.0001$)	6880
Microorganism	5029
Prokaryotic microorganism	4913
Eukaryotic microorganism	2
Eukaryotic viruses and phages	118
Animal	1748
Protozoa	3
Metazoa	1745
Parasite	17
Insects and Vertebrates	1728
Plant (Alga)	17
Unknown	81

Table S5. Contigs related to prokaryotic microorganism as determined with Blastx and the GenBank database.

Family	Name	Contig number
Acinetobacter	*Acinetobacter* sp.	2
Clostridium	*Clostridium difficile*	544
	Clostridium sp.	159
	Clostridium sticklandii	28
	Coprococcus comes	1
	Coprococcus eutactus	1
Corynebacterium	*Corynebacterium efficiens*	1
Enterococcus	*Enterococcus faecalis*	3
	Enterococcus saccharolyticus	1
Flavobacteria	*Flavobacteria bacterium*	2
Francisella	*Francisella philomiragia* subsp. philomiragia	1
Fusobacterium	*Fusobacterium necrophorum*	1
Gemella	*Gemella haemolysans*	3
	Gemella morbillorum	2
	Gemella sanguinis	1
Haemophilus	*Haemophilus parainfluenzae*	57
	Haemophilus influenzae	17
Klebsiella	*Klebsiella pneumoniae*	12
Micrococcus	*Micrococcus luteus*	19
Mycoplasma	*Mycoplasma fermentans*	1
Paenibacillus	*Paenibacillus* sp.	3
	Paenibacillus popilliae	1
Streptococcus	*Streptococcus infantis*	18
	Streptococcus mutans	15
	Streptococcus equinus	12
	Streptococcus gallolyticus	8
	Streptococcus bovis	3
	Streptococcus equi ssp equi	2
	Staphylococcus chromogenes	1
	Streptococcus alactolyticus	1

Table S5. *Cont.*

Family	Name	Contig number
Helicobacter	*Heliobacterium modesticaldum*	9
	Helicobacter mustelae	2
Aggregatibacter	*Aggregatibacter segnis*	3
Aspergillus	*Aspergillus niger*	2
	Aspergillus nidulans	1
	Aspergillus oryzae	1
Bacillus	*Bacillus*	6
	Bacillus stratosphericus	3
	Bacillus thuringiensis	1
Bacteroides	*Bacteroides uniformis*	2
	Bacteroides caccae	1
	Bacteroides fragilis	1
	Bacteroides ovatus	1
	Bacteroides vulgatus	1
Beggiatoa	*Beggiatoa*	2
Bergeyella	*Bergeyella zoohelcum*	2
Bifidobacterium	*Bifidobacterium adolescentis*	1
Brevibacillus	*Brevibacillus laterosporus*	5
Citrobacter	*Citrobacter koseri*	5
	Citrobacter rodentium	3
	Citrobacter	2
	Citrobacter youngae	1
Clostridium	*Clostridium butyricum*	187
	Clostridium bartlettii	100
	Clostridium celatum	75
	Clostridium beijerinckii	35
	Clostridium sp. Maddingley	29
	Clostridium saccharoperbutylacetonicum	23
	Clostridium carboxidivorans	7
	Clostridium sporogenes	7
	Clostridium cellulovorans	6
	Clostridium ultunense Esp	6
	Clostridium acetobutylicum	5
	Clostridium clostridioforme	5
	Clostridium leptum	4
	Clostridium kluyveri	3
	Clostridium arbusti	2
	Clostridium ljungdahlii	2
	Clostridium polysaccharolyticum	2
	Clostridium acidurici	1
	Clostridium bolteae	1
	Clostridium botulinum	1
	Clostridium clariflavum	1
	Clostridium pasteurianum	1
	Clostridium roseum	1
	Clostridium sp.	1
	Clostridium symbiosum	1
	Clostridium thermocellum	1

Table S5. *Cont.*

Family	Name	Contig number
Collinsella	*Collinsella*	2
Deinococcus	*Deinococcus radiodurans*	2
Escherichia	*Escherichia Coli*	873
Elizabethkingia	*Elizabethkingia meningoseptica*	1
Enterobacter	*Enterobacter cloacae*	6
	Enterobacter hormaechei	1
	Enterobacteriaceae bacterium	1
Erysipelotrichaceae	*Erysipelotrichaceae*	3
Eubacteriaceae	*Eubacteriaceae bacterium*	3
	Eubacterium dolichum	2
	Eubacterium limosum	1
	Eubacterium siraeum	1
	Eubacterium ventriosum	1
Finegoldia	*Finegoldia magna*	1
Fusobacterium	*Fusobacterium gonidiaformans*	1
	Fusobacterium nucleatum subsp. vincentii	1
	Fusobacterium varium	1
Gordonia	*Gordonia amarae*	1
Granulicatella	*Granulicatella elegans*	11
	Haemophilus somnus	1
Hafnia	*Hafnia alvei*	1
Halobacillus	*Halobacillus sp.*	1
Halomanas	*Halomonas titanicae*	1
Helicobacter	*Helicobacter cinaedi*	2
	Helicobacter hepaticus	1
	Helicobacter pullorum	1
Lachnospiraceae	*Lachnospiraceae bacterium*	6
	Lachnospiraceae oral taxon	1
Lactobacillu	*Lactobacillus jensenii*	6
	Lactobacillus rhamnosus	3
	Lactobacillus fermentum	2
	Lactobacillus brevis	1
	Lactobacillus casei	1
	Lactobacillus curvatus	1
	Lactobacillus pentosus	1
	Lactobacillus saerimneri	1
	Lactobacillus salivarius	1
Leptotrichia	*Leptotrichia goodfellowii*	2
	Leptotrichia hofstadii	1
Listeria	*Listeria grayi*	1
Magnetospirillum	*Magnetospirillum gryphiswaldense*	5
Methanobrevibacter	*Methanobrevibacter smithii*	1
Neisseria	*Neisseria polysaccharea*	30
Peptoniphilus	*Peptoniphilus indolicus*	2
Peptostreptococcus	*Peptostreptococcus stomatis*	5

Table S5. *Cont.*

Family	Name	Contig number
Photorhabdus	*Photorhabdus asymbiotica*	2
	Photorhabdus asymbitotica	1
Planctomyces	*Planctomyces*	1
Propionibacterium	*Propionibacterium acidipropionici*	3
	Propionibacterium acidipropionici	1
Ruminococcus	*Ruminococcus obeum*	6
	Ruminococcus gnavus	2
Scheffersomyces	*Scheffersomyces stipitis*	2
Selenomonas	*Selenomonas noxia*	1
Staphylococcus	*Staphylococcus epidermidis*	1
Stigmatella	*Stigmatella aurantiaca*	1
Streptococcus	*Streptococcus mitis*	11
	Streptococcus oralis	10
	Streptococcus tigurinus	9
	Streptococcus sanguinis	7
	Streptococcus thermophilus	7
	Streptococcus macacae	5
	Streptococcus australis	4
	Streptococcus ictaluri	4
	Streptococcus macedonicus	4
	Streptococcus macedonicus	4
	Streptococcus parasanguinis	4
	Streptococcus pasteurianus	4
	Streptococcus ratti	4
	Streptococcus sobrinus	4
	Streptococcus criceti	2
	Streptococcus criceti	2
	Streptococcus constellatus ssp constellatus	1
Streptomyces	*Streptomyces* sp.	36
Thermoanaerobacter	*Thermoanaerobacter ethanolicus*	1
	Thermoanaerobacter mathranii	1
	Thermoanaerobacter tengcongensis	1
	Thermoanaerobacterium thermosaccharolyticum	1
	Thermoanaerobacterium xylanolyticum	1
Veillonella	*Veillonella* spp.	2
Vivrio	*Vivrio mimicus*	1
Yersinia	*Yersinia frederiksenii*	1
Yokenella	*Yokenella regensburgei*	1
Ajellomyces	*Ajellomyces capsulatus*	2
Ureaplasma	*Ureaplasma parvum serovar*	2
Thioflavicoccus	*Thioflavicoccus mobilis*	1
Kurthia	*Kurthia* sp.	1
Cyanothece	*Cyanothece* sp.	1
Chlorobaculum	*Chlorobaculum tepidum*	1
Deinococcus	*Deinococcus radiodurans*	2

Table S5. *Cont.*

Family	Name	Contig number
Wolinella	*Wolinella succinogenes*	1
Exophiala	*Exophiala dermatitidis*	2
Prevotella	*Prevotella* sp.	1
Penicillium	*Penicillium chrysogenum Wisconsin*	2
Desulfotomaculum	*Desulfotomaculum gibsoniae*	3
Desulfovibrio	*Desulfovibrio magneticus*	1
Dichomitus squalens	*Dichomitus squalens*	1
mixed culture bacterium	mixed culture bacterium	2
uncultured	uncultured Acidobacteria bacterium	4
uncultured	uncultured alpha proteobacterium	1
uncultured	uncultured bacterium	1
uncultured	uncultured beta proteobacterium	2
uncultured	uncultured *Chromatiales bacterium*	3
uncultured	uncultured *Desulfobacterales bacterium*	1
uncultured	uncultured *Desulfobacterium* sp.	1
uncultured	uncultured gamma proteobacterium	3
uncultured	uncultured *Oceanospirillales bacterium*	1
uncultured	uncultured *Rhodobacterales bacterium*	1
Caenorhabditis	*Caenorhabditis remanei*	1
Bacillus	*Bacillus macauensis*	1
Geobacillus	*Geobacillus thermoleovorans*	1
Sporolactobacillus	*Sporolactobacillus vineae*	1
Acidaminococcus fermentans	*Acidaminococcus fermentans*	1
Actinobacillus	*Actinobacillus pleuropneumoniae*	1
	Actinomyces viscosus	2
Aggregatibacter	*Aggregatibacter actinomycetemcomitans*	4
Arthrobacter	*Arthrobacter citreus*	1
Aspergillus	*Aspergillus flavus*	1
Bacillus	*Bacillus cereus*	5
	Bacillus licheniformis	2
	Bacillus bataviensis	1
	Bacillus halodurans	1
	Bacillus mycoides	1
Brachyspira	*Brachyspira hyodysenteriae*	2
Campylobacter	*Campylobacter jejuni ssp jejuni*	3
Chryseobacterium	*Chryseobacterium gleum*	2
Ciona	*Ciona intestinalis*	1
Clostridium	*Clostridium perfringens*	1526
	Clostridium hiranonis	70
	Clostridium botulinum	52
	Clostridium lentocellum	13
	Clostridium novyi	3
	Clostridium tetani	3
	Clostridium septicum	2
	Clostridium termitidis	2

Table S5. *Cont.*

Family	Name	Contig number
Edwardsiella	*Edwardsiella tard*	3
Enterobacter	*Enterobacter hormaechei*	1
Enterococcus	*Enterococcus casseliflavus*	1
Erwinia	*Erwinia amylovory*	4
Escherichia	*Escherichia fergusonii*	10
Gallibacterium	*Gallibacterium anatis*	2
Haemophilus	*Haemophilus haemolyticus*	7
Helicobacter	*Helicobacter bilis*	4
	Helicobacter pylori	1
higella	*higella flexneri*	92
Klebsiella	*Klebsiella oxytoca*	5
Lactococcus	*Lactococcus garvieae*	2
Lawsonia	*Lawsonia intracellularis*	4
Legionella	*Legionella pneumophila subsp. Pneumophila*	2
Listeria	*Listeria monocytogenes*	2
Mycobacterium	*Mycobacterium tuberculosis*	1
Magnaporthe	*Magnaporthe oryzae*	5
Mannheimia	*Mannheimia haemolytica*	4
	Mannheimia succiniciproducens	1
Marinobacter	*Marinobacter hydrocarbonoclasticus*	3
Melissococcus	*Melissococcus plutonius*	1
Metarhizium	*Metarhizium anisopliae*	1
Methanococcus	*Methanococcus maripaludis*	2
	Methanococcus voltae	1
Mycoplasma	*Mycoplasma gallisepticum*	1
Myxococcus	*Myxococcus* sp.	1
Neisseria	*Neisseria meningitidis*	3
	Neisseria subflava	1
Paenibacillus	*Paenibacillus alvei*	2
Paracoccidioides	*Paracoccidioides brasiliensis*	2
Pasteurella	*Pasteurella multocida*	6
Pediococcus	*Pediococcus acidilactici*	2
Peptostreptococcus	*Peptostreptococcus anaerobius*	3
Providencia	*Providencia stuartii*	1
Psychrobacter	*Psychrobacter cryohalolentis*	1
Rhodococcus	*Rhodococcus equi*	1
Rhodospirillum	*Rhodospirillum photometricum*	6
Riemerella	*Riemerella anatipestifer*	1
Saccharomonospora	*Saccharomonospora paurometabolica*	1
Salmonella	*Salmonella enterica ssp enterica*	128
	Salmonella enterica ssp arizonae	2
	Salmonella bongori	1
	Salmonella enterica subsp. houtenae str	1
	Salmonella paratyphi	1
	Salmonella typhimurium	1

Table S5. *Cont.*

Family	Name	Contig number
Shigella	*Shigella dysenteriae*	26
	Shigella sonnei	16
	Shigella boydii	13
	Shigella spp.	2
Staphylococcus	*Staphylococcus aureus*	5
Streptococcus	*Streptococcus agalactiae*	30
	Streptococcus pneumoniae	17
	Streptococcus anginosus	14
	Streptococcus suis	14
	Streptococcus dysgalactiae ssp dysgalactiae	11
	Streptococcus pyogenes	10
	Streptococcus iniae	6
	Streptococcus uberis	6
	Streptococcus equi ssp zooepidemicus	5
	Streptococcus porcinus	5
	Streptococcus salivarius	5
	Streptococcus vestibularis	5
	Streptococcus intermedius	3
	Streptococcus canis	1
Streptomyces	*Streptomyces ghanaensis*	4
	Streptomyces coelicoflavus	3
	Streptomyces albus	2
	Streptomyces roseosporus	1
Talaromyces	*Talaromyces stipitatus*	4
Trichoderma	*Trichoderma harzianum*	1
Vibrio	*Vibrio cholerae*	3
Weissella	*Weissella*	1
Yersinia	*Yersinia pestis*	2
Marssonina	*Marssonina brunnea f. sp*	1
Serratia	*Serratia plymuthica*	1
Total		4913

Table S7. Gammaretroviruses used in the phylogenetic analyses.

Virus	Abbreviation	ID	Host	Reference
Rhinolophus pusillus retrovirus	RpuRV	JQ292909	bat	1
R. pearsoni retrovirus	RpeRV	JQ292914	bat	1
R. megaphyllus retrovirus	RmRV	JQ292911	bat	1
R. affinis retrovirus	RaRV	JQ292913	bat	1
Myotis ricketti retrovirus	MrRV	JQ292912	bat	2
Pteropusalecto retrovirus	PaRV	JQ292910	bat	1
Megaderma lyra retrovirus	MlRV	JQ951956	bat	2
Rousettus leschenaultia retrovirus	RlRV	JQ951957	bat	2

Table S7. *Cont.*

Virus	Abbreviation	ID	Host	Reference
R. ferrumequinum retrovirus	RfRV	JQ303225	bat	1
Reticuloendotheliosis virus	REV	NC_006934	bird	3
Friend murine leukemia virus	F-MuLV	NC_001362	mouse	4
Moloney murine leukemia virus	M-MuLV	NC_001501	mouse	5
Murinetype C retrovirus	M-CRV	NC_001702	mouse	
Porcineendogenous type C retrovirus class A	PERV-A	AJ293656	pig	6
Porcineendogenous type C retrovirus class B	PERV-B	AY099324	pig	7
Porcineendogenous type C retrovirus class C	PERV-C	EF133960	pig	8
Mus dunni endogenous virus	MDEV	AF053745	mouse	9
RD114 retrovirus	RD114	NC_009889	cat	
Koala retrovirus	KoRV	AF151794	koala	10
Baboon endogenous virus	BaEV	D10032	nonhuman primates	11
Xenotropic murine leukemia virus	X-MuLV	AEI59728	mouse	12
Porcineendogenous retrovirus	PERV	CAA76582.1	pig	13
Orcinus orca endogenous retrovirus	OOEV	GQ222416	whale	14

Reference

1. Cui, J.; Tachedjian, M.; Wang, L.; Tachedjian, G.; Wang, L.-F.; Zhang, S. Discovery of retroviral homologs in bats: Implications for the origin of mammalian gammaretroviruses. *J. Virol.* **2012**, *86*, 4288–4293.
2. Cui, J.; Tachedjian, G.; Tachedjian, M.; Holmes, E.C.; Zhang, S.; Wang, L.-F. Identification of diverse groups of endogenous gammaretroviruses in mega-and microbats. *J. Gen. Virol.* **2012**, *93*, 2037–2045.
3. Wang, Y.; Cui, Z.; Jiang, S. Sequence analysis for the complete proviral genome of reticuloendotheliosis virus Chinese strain HA9901. *Sci. China C* **2006**, *49*, 149–157.
4. Perryman, S.; Nishio, J.; Chesebro, B. Complete nucleotide sequence of Friend murine leukemia virus, strain FB29. *Nucleic Acids Res.* **1991**, *19*, 6950.
5. Shinnick, T.M.; Lerner, R.A.; Sutcliffe, J.G. Nucleotide sequence of moloney murine leukaemia virus. *Nature* **1981**, *293*, 543–548.
6. Krach, U.; Fischer, N.; Czauderna, F.; Tönjes, R.R. Comparison of replication-competent molecular clones of porcine endogenous retrovirus class A and class B derived from pig and human cells. *J. Virol.* **2001**, *75*, 5465–5472.
7. Bartosch, B.; Weiss, R.A.; Takeuchi, Y. PCR-based cloning and immunocytological titration of infectious porcine endogenous retrovirus subgroup A and B. *J. Gen. Virol.* **2002**, *83*, 2231–2240.
8. Ma, Y.; Lv, M.; Xu, S.; Wu, J.; Tian, K.; Zhang, J. Identification of full-length proviral DNA of porcine endogenous retrovirus from Chinese Wuzhishan miniature pigs inbred. *Comp. Immunol. Microbiol. Infect. Dis.* **2010**, *33*, 323–331.
9. Wolgamot, G.; Bonham, L.; Miller, A.D. Sequence analysis of Mus dunni endogenous virus reveals a hybrid VL30/gibbon ape leukemia virus-like structure and a distinct envelope. *J. Virol.* **1998**, *72*, 7459–7466.

10. Hanger, J.J.; Bromham, L.D.; McKee, J.J.; O'Brien, T.M.; Robinson, W.F. The nucleotide sequence of koala (*Phascolarctos cinereus*) retrovirus: A novel type C endogenous virus related to Gibbon ape leukemia virus. *J. Virol.* **2000**, *74*, 4264–4272.
11. Kato, S., Matsuo, K., Nishimura, N., Takahashi, N., Takano, T. The entire nucleotide sequence of baboon endogenous virus DNA: A chimeric genome structure of murine type C and simian type D retroviruses. *Jpn. J. Genet.* **1987**, *62*, 127–137.
12. Sfanos, K.S.; Aloia, A.L.; Hicks, J.L.; Esopi, D.M.; Steranka, J.P.; Shao, W.; Sanchez-Martinez, S.; Yegnasubramanian, S.; Burns, K.H.; Rein, A. Identification of replication competent murine gammaretroviruses in commonly used prostate cancer cell lines. *PLoS One* **2011**, *6*, e20874.
13. Czauderna, F.; Fischer, N.; Boller, K.; Kurth, R.; Tönjes, R.R. Establishment and characterization of molecular clones of porcine endogenous retroviruses replicating on human cells. *J. Virol.* **2000**, *74*, 4028–4038.
14. LaMere, S.A.; Leger, J.A.S.; Schrenzel, M.D.; Anthony, S.J.; Rideout, B.A.; Salomon, D.R. Molecular characterization of a novel gammaretrovirus in killer whales (*Orcinus orca*). *J. Virol.* **2009**, *83*, 12956–12967.
